# A case of limbic encephalitis associated with severely COVID-19 infection

**DOI:** 10.1016/j.amsu.2022.103274

**Published:** 2022-01-31

**Authors:** Amine Elmouhib, Hicham Benramdane, Fatima zohra Ahsayen, Inass Arhoun El Haddad, Abdelilah El Ghalet, Ilyas Laaribi, Houssam Bkiyar, Siham Nasri, Imane Skiker, Brahim Housni

**Affiliations:** a Department of Intensive Care Unit, Mohammed VI University Hospital, Oujda, Morocco; b Department of Radiology, Mohammed VI University Hospital, Oujda, Morocco; c Faculty of Medicine and Pharmacy, Mohammed 1 st University, Oujda, Morocco; d Mohammed First University Oujda, FMP Oujda, LAMCESM, Oujda, Morocco

**Keywords:** Severely covid-19 infection, Limbic system, Limbic encephalitis, Post-infectious encephalitis

## Abstract

**Introduction:**

SARS Cov-2 infection is a pandemic that continues to ravage the world. The list of its complications continues to grow every day.

**Case presentation:**

We report the case of a young patient admitted to intensive care for limbic encephalitis associated with severely COVID-19 infection.

**Discussion:**

With the COVID-19 outbreak being a global pandemic, various neurological manifestations have been reported. On the other hand, diverse cases of limbic encephalitis related to COVID-19 have been recently described, they are related either to hyper inflammation syndrome with massive release of inflammatory cytokines or to secondary autoimmune response.

**Conclusion:**

Seriously ill COVID-19 patients are at a higher risk of limbic encephalitis. It is therefore important to monitor Neurological Events in COVID-19 patients. This makes it possible to start the appropriate treatments quickly and avoid complications.

## Highlights

• Several studies have demonstrated an association between SARS-CoV-2 and encephalitis event.
• Limbic encephalitis complication is usually associated with severe respiratory symptoms of COVID-19.
• Delayed onset of neurological symptoms compared to respiratory involvement, with a median of two weeks.
• Limbic encephalitis is a serious neurological complication of SARS-CoV-2 infection.

## 1 Introduction

In December 2019, a new coronavirus responsible for a disease called SARS-CoV-2 (severe acute respiratory syndrome coronavirus 2) appeared in Wuhan, China [[1], [2], [3], [4]]. It quickly spread throughout the world, despite the drastic containment measures and sacrifices put in place by several countries. The new SARS-CoV-2 is primarily caused by respiratory symptoms [5].

Several neurological complications of the disease have been reported, including encephalopathy, meningoencephalitis, strokes, seizures and myalgia [6]. In this article, we present a case of post SARS Cov-2 limbic encephalitis. This suggests that this virus has high inflammatory potential as well as autoimmune.

## 2 Presentation of the clinical case

We report in this work the case of a patient hospitalized in intensive care for a severe SARS Cov-2 infection who presented during her evolution a consciousness disorder secondary to limbic encephalitis.

The patient was a 54-year-old woman, vaccinated by Astra Zeneca, without any notion of taking toxic, no significant pathological history, nor any genetic and psychosocial antecedents, admitted on September 12, 2021 to the intensive care for the management of severe respiratory distress.

At admission the patient was stable on both neurological (a Glasgow Coma Score of 15/15) and hemodynamic (normo tense with blood pressure 125/65 mmHg, normo carde at 98 bpm) states, while on respiratory level, he was unstable with an oxygen saturation rate of 79% and a breathing rate of 27 breaths/min.

Before its desaturation the patient was put on High Throughput Oxygenation (FiO2 = 40%; Throughput = 50l/min) with a SpO2 = 92%.

An arterial blood gas test revealed a pH of 7.31, PO2: 60, PCO2: 55.5, SpO2: 92% on High Flow Oxygenation (FiO2 = 40%; Flow = 50l/min).

**Fig. 1:**
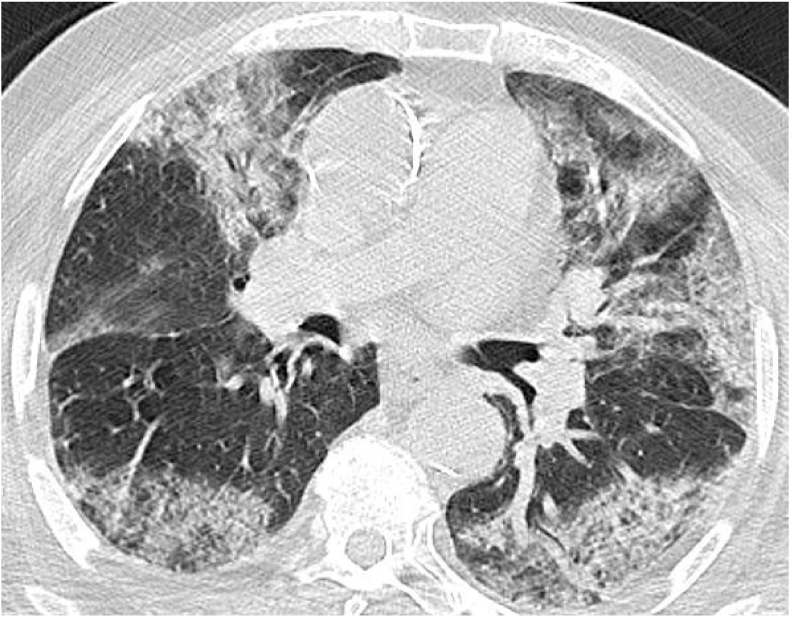
Axial no enhanced high-resolution CT (lung window) demonstrates extensive ground-glass opacities in a peripheral and central distribution, typical appearance of COVID-19 pneumonia, with more than 75% of the lung involved.

A chest CT was also performed showing diffuse pure ground-glass opacities, typical appearance of COVID-19 pneumonia, classified CORADS 5 with a critical extended parenchymal involvement greater than 75% [Fig. 1].

A PCR (polymerase chain reaction) test on a nasopharyngeal sample was positive and a positive serology SARS-CoV-2 (IgG, IgM positive).

The biological balance was in favor of a lymphopenia 360 cells/microl, hemoglobin at 12.4g/dl, platelet count at 523,000 cells/microl, leukocytosis at 12,000 cells/microl, CRP at 200mg/l with a negative PCT at 0.05 μg/l, ferritin at 2300 μg/l and a dosage of interleukin 6 at 2360pg/ml, D-dimers at 5.32mg/l with high levels of fibrinogen at 12g/l, normal ionogram with natremia at 138 meq/l, kalemia at 4 meq/l, calcemia corrected at 95mg/l, kidney and liver function were normal.

The patient was given vitamin C 1000mg 1 cp x2/day, zinc 45mg 1cp/day, curative anticoagulation with enoxaparin 0.6 cc/12h, Tocilizumab 600 mg single dose, ceftriaxone 2g/day + Levofloxacin 500mgx2/day and Dexamethasone 6mg/day.

12 days after hospitalization the patient presented with altered consciousness associated with fever, a progressive worsening of dyspnea and oxygen requirements.

Clinical examination showed a Glasgow Coma Score of 11/15 (eye opening 4/4, motor response 4/6 verbal response 3/5), and neurological evaluation revealed hypotonia of the 4 limbs with depressed reflexes in the lower limbs.

The patient was hemodynamically stable, normo tense with blood pressure 134/68 mmHg, normo carde at 90 bpm, SpO2 = 91% on High Throughput Oxygenation (FiO2 = 75%; Throughput = 60l/min) as well as fever up to 39.5°with capillary glucose at 1.12g/l.

**Fig. 2:**
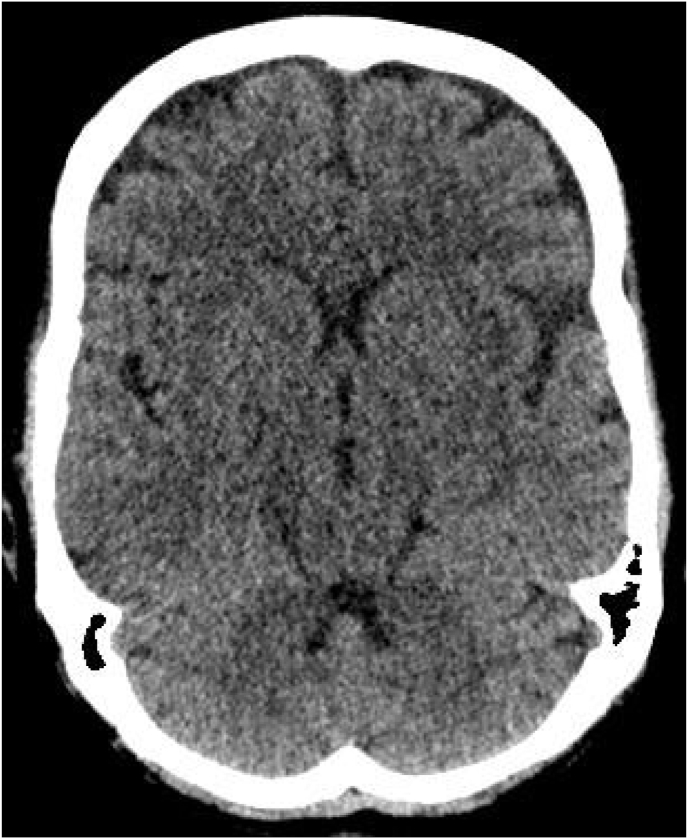
Normal axial non-contrast CT of brain.

In front of his disorder of consciousness, a brain scan was carried out returning normal [Fig. 2].

Examination of the cerebrospinal fluid (CSF) showed the white blood cell count 3/mm3, red blood cell count 11/mm3, protein level 1.0g/l (0.15–0.45) and glucose level 3.5mmol/l (2.2–3.9).

A biological control check-up was performed showing a worsening of the inflammatory check-up, while ionogram, kidney and liver function were normal.

**Fig. 3:**
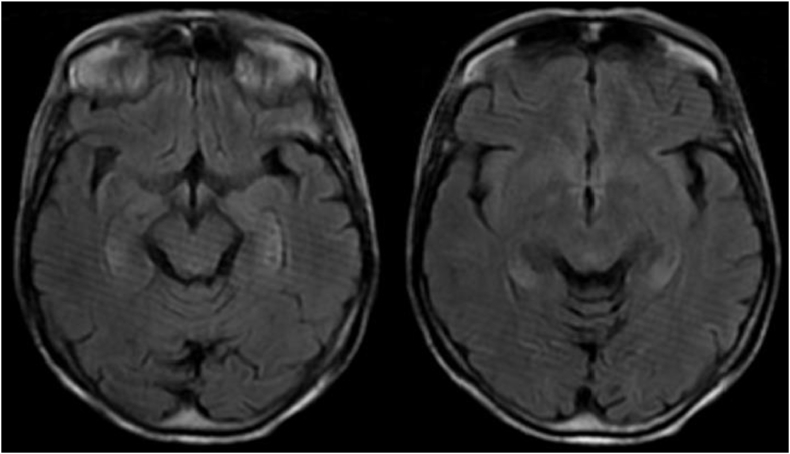
Axial T2-weighted FLAIR imaging demonstrate bilateral temporal hyper intensity.

**Fig. 4:**
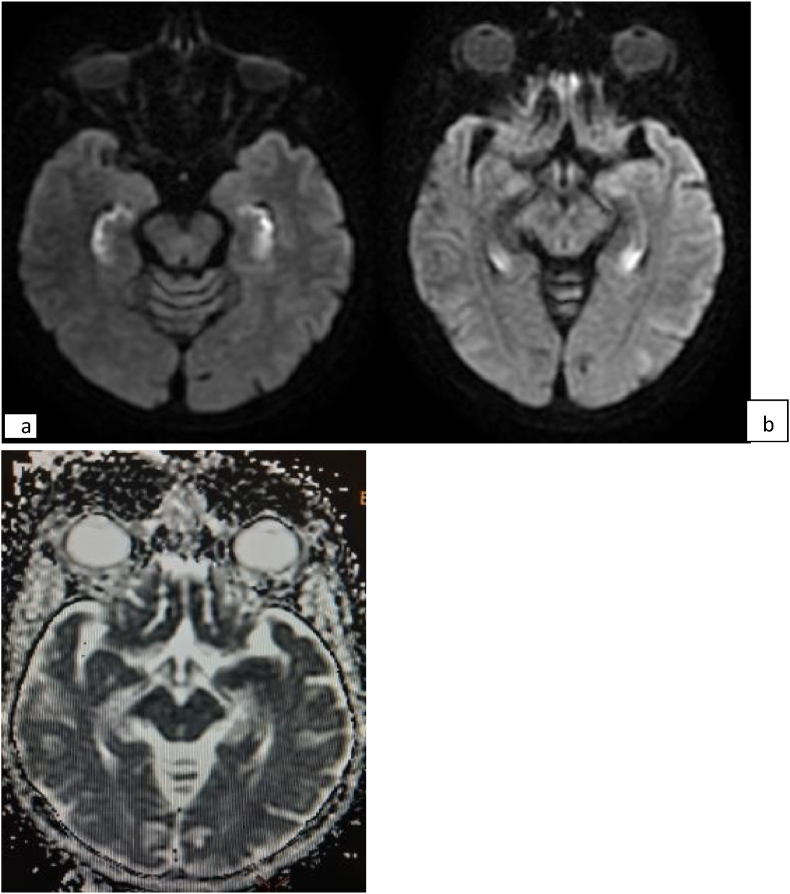
DWI shows diffuse bilateral hippocampal diffusion hyper intensity (a), without diffusion restriction on ADC map (b).

Brain MRI (Magnetic resonance imaging) showed high-signal intensity lesion on diffusion-weighted imaging (DWI), T2 fluid-attenuated inversion recovery (FLAIR) in the temporal lobes, without diffusion restriction on apparent diffusion coefficient (ADC) map [Fig. 3, Fig. 4].

A PCR test of the LCR highlighted the Ag of the SARS cov2.

Therapeutically, we put the patient on anti-comitial sodium valproate treatment: Depakin 500mg/8h, and high-dose of methylprednisolone.

With a spectacular response to corticosteroids, the evolution was favorable after 20 days with a clear improvement on the respiratory and neurological plane. We also noted an improvement in lymphopenia and inflammatory assessment. The patient was transferred to the neurology ward and returned home after 2 weeks in hospital.

This case is written following the SCARE guidelines [7].

## 3 Discussion

Limbic encephalitis is one of neurological complications in patients hospitalized with Covid-19, the brain consequences of SARS-CoV-2, whether direct or indirect, are not yet fully understood [8]. The virus by causing endothelial aggression of the cerebral vessels could participate in this encephalopathy, by weakening the blood-brain barrier which facilitates the passage of pro-inflammatory cytokines [3]. Faced with the multiple pathophysiological mechanisms that can explain this encephalopathy, the debate remains open.

Several cases of COVID-19-related encephalitis have been recently described; the majorities are related to secondary hyper inflammation syndrome with massive release of cytokines and chemokine [9,10].

However, rare cases of encephalitis have been attributed to an autoimmune mechanism via antibodies directed against the surface of neuronal cells or synaptic proteins triggered by the virus, a true Para/post-infectious disease [3].

Our patient and other cases of limbic encephalitis with severe respiratory symptoms of COVID-19 have had a delayed onset of neurological symptoms compared to respiratory impairment, with a median of two weeks.

Future studies aimed at identifying biomarkers could help to correctly identify physio-pathological mechanisms underlying the broad spectrum of encephalitis and to better clarify the pathological process of limbic encephalitis in the context of COVID-19.

## 4 Conclusion

Very limited cases of autoimmune limbic encephalitis have been described. It is therefore crucial to monitor neurological events in COVID-19 patients especially in the event of serious impairment and think about limbic encephalitis when an altered mental state appears late in the course of the disease. This makes it possible to quickly start the appropriate treatments and avoid complications.

## Ethical approval

This is a case report that does not require a formal ethical committee approval. Data were anonymously registered in our database. Access to data was approved by the head of the department.

## sources of funding

This research was not funded

## Author contribution

Dr Elmouhib Amine and Dr. Hicham Benramdane: are principal investigators that collected and analyzed data, wrote the manuscript and prepared the final draft for the submission. Prof. Brahim Housni and Prof. Imane Skiker: supervised the research project and approved the final draft for publication. All authors approved the final version of the manuscript.

## Registration of research studies

This is not an interventional study. We only reported the patients’ findings from our database as a case series.

## Guarantor

Dr Elmouhib Amine.

## Consent

Written informed consent was obtained from the patient for publication of this case report and accompanying images. A copy of the written consent is available for review by the Editor-in-Chief of this journal on request.

## Declaration of competing interest

The authors declare no conflict of interest.
